# Establishing Local Diagnostic Reference Levels for Head Computed Tomography Examinations

**DOI:** 10.3390/biomedicines12112446

**Published:** 2024-10-25

**Authors:** Sandra Modlińska, Marcin Rojek, Michał Bielówka, Jakub Kufel

**Affiliations:** 1 Department of Radiodiagnostics, Invasive Radiology and Nuclear Medicine, Faculty of Medical Sciences in Katowice, Medical University of Silesia, 40-752 Katowice, Poland; smodlinska@sum.edu.pl; 2 Department of Radiology and Nuclear Medicine, Faculty of Medical Sciences in Katowice, Medical University of Silesia, 40-752 Katowice, Poland; jakubkufel92@gmail.com; 3 Students’ Scientific Association of Computer Analysis and Artificial Intelligence, Department of Radiology and Nuclear Medicine, Medical University of Silesia, 40-752 Katowice, Poland; marcin.arojek@gmail.com

**Keywords:** diagnostic reference levels, DRL, head CT scans, radiation dose optimization

## Abstract

**Background**/**Objectives**: Head Computed Tomography (CT) is an essential diagnostic tool for identifying brain pathologies and visualizing blood vessels. However, CT exposes patients to ionizing radiation, making it necessary to establish local diagnostic reference levels (DRLs) to ensure patient safety. This study aimed to establish DRLs for head CT scans and assess the influence of patient characteristics on radiation dose. **Methods**: A retrospective analysis was conducted on 2043 non-contrast and 488 contrast-enhanced head CT scans performed between 1 July 2023 and 31 March 2024 using a SIEMENS SOMATOM Definition Edge machine. Computed Tomography Dose Index (CTDIvol) and Dose-Length Product (DLP) values were analyzed, with DRLs set at the 75th percentile. The influence of gender, height, and weight on radiation dose was also evaluated. **Results**: The DRL for both non-contrast and contrast-enhanced scans was 58.18 mGy for CTDIvol and 1018.11 mGy·cm for DLP per acquisition. Total DLP was 2046.09 mGy·cm for contrast-enhanced and 1027.99 mGy·cm for non-contrast scans. No significant correlation was found between patient characteristics and radiation dose, allowing for a uniform DRL to be established. **Conclusions**: Uniform DRLs were successfully established for head CT scans, ensuring safe radiation doses for both non-contrast and contrast-enhanced studies. The lack of correlation between patient-specific factors and dose supports the use of standardized DRLs, contributing to optimized radiation safety in head CT diagnostics.

## 1. Introduction

In Poland, head CT scans are among the most commonly available and performed examinations in hospitals and specialized diagnostic centers. This method allows for obtaining detailed images of structures inside the skull–brain structures as well as blood vessels. The introduction of these examinations into routine clinical practice enables rapid and precise identification of various pathologies and facilitates effective therapy.

Although CT plays a significant role in disease treatment, it is responsible for the risk of developing radiation-induced tumors, as CT uses ionizing radiation to generate images of the examined body [1,2]. Erdal Karavas et al. emphasize that ionizing radiation from medical imaging, particularly CT scans, has nearly doubled in the last two decades, primarily increasing concerns over cancer risks. Ionizing radiation can damage DNA, raising an individual’s lifetime risk of cancer, especially with repeated exposure [3]. The study conducted by Erdem Fatihoglu et al. discusses the impact of unnecessary imaging, such as chest CT scans, which not only increases healthcare costs but also exposes patients to potentially harmful ionizing radiation. This exposure, particularly from repetitive scans, is associated with increased long-term risks, including cancer, with children and young adults being especially vulnerable [4].

The amount of radiation dose administered to patients is associated with various factors [5]. Medical personnel pay special attention to ensuring optimal benefits with minimal risk to the patient by establishing appropriate Diagnostic Reference Levels (DRLs), which define the threshold values of ionizing radiation doses according to which examinations should be conducted.

To establish DRLs, a retrospective analysis of a wide range of head CT examinations performed within a specified period was conducted. These examinations included both procedures with and without contrast. It is important to consider potential differences in dosage associated with the necessity of contrast administration, which may lead to increased radiation dose for the patient [6].

The International Commission on Radiological Protection (ICRP) first introduced the term ‘diagnostic reference level’ (DRL) in 1996. Subsequently, this concept was developed, and practical guidelines were presented in 2001. The DRL has been proven to be an effective tool that aids in the optimization of protection in the medical exposure of patients for diagnostic and interventional procedures [7].

This study presents the results of the analysis and obtained local diagnostic levels for head CT examinations with and without contrast, conducted at a university hospital in Poland, taking into account gender and physical differences of patients. The aim is to provide guidelines for optimal radiation dosing, following current standards and recommendations, which are of significant importance for the safety and diagnostic effectiveness of CT procedures.

## 2. Materials and Methods

The retrospective analysis covered examinations conducted from 1 July 2023, to 31 March 2024. The analysis included head CT scans performed according to the Head^GLOWA_SEQ (Adult) protocol using a Siemens SOMATOM Definition Edge CT scanner (Siemens Healthineers, Erlangen, Germany). The examination conditions were set as follows: 120 kV, 370 mA, slice thickness: 0.6 mm. The acquisition range in all examinations was the same: from the base of the skull to the vertex. All contrast-enhanced scans were performed after the administration of 40 mL of the positive contrast agent Omnipaque 350 (GE Healthcare, Chicago, IL, USA), thus the variable was omitted from the analysis. In total, there were 2043 non-contrast examinations and 488 contrast-enhanced examinations.

Local Diagnostic Reference Levels (DRLs) were based on two fundamental values used in CT: CTDIvol and Dose Length Product (DLP), and were calculated as the 75th percentile of both these values. The CTDI (Computed Tomography Dose Index) is a key parameter for measuring radiation dose during CT scanning. It quantifies the radiation exposure to a single slice of tissue from a single rotation of the CT scanner, expressed in milligrays (mGy). Specifically, CTDIvol is used to reflect the average dose per volume for the entire scan, considering both the pitch factor (distance covered per rotation) and the slice thickness. Additionally, the CTDIvol w/acq value, which represents the CTDIvol value per acquisition phase excluding the CTDIvol value from the topogram, was also analyzed.

For each of the analyzed examinations, the values of DLP and CTDIvol, for each phase and for the entire examination, as well as CTDIvol w/acq, were obtained from the Dose&Care dose monitoring system by Guerbet (Roissy, France). Based on the number of phases performed and information about contrast agent administration, the examinations were divided into two groups: CT scans with contrast and CT scans without contrast.

For both types of examinations, local DRLs were determined depending on the patient’s gender in the reference patient group (height of 170 cm +/− 5 cm and weight of 70 kg +/− 5 kg). The total number of reference patients was 1385 women and 1141 men. Local reference levels for contrast-enhanced and non-contrast CT heads were counted in the following groups: all women (regardless of weight and height), reference female patients, all men (regardless of weight and height), reference male patients, reference patients regardless of gender, all patients. All statistical analyses were conducted in the Python environment using the matplotlib, pandas, and scipy libraries, while matplotlib and plotly were used for graphical representations. A significance level of *p* < 0.05 was considered statistically significant.

To examine the influence of patient weight and height on the DRL values for contrast-enhanced head CT scans and non-contrast head CT scans. DRL values were calculated independently of the patient’s physical parameters, both by gender and for the overall DRL value, for all conducted examinations. Subsequently, it was investigated whether there was a statistically significant difference between the obtained DRL values for contrast-enhanced scans and non-contrast scans.

## 3. Results

Based on the conducted analysis, local Diagnostic Reference Levels (DRLs) for head CT scans were determined. The results for CT scans with contrast are presented in Table 1, and the results for CT scans without contrast are presented in Table 2.

**Figure 1:**
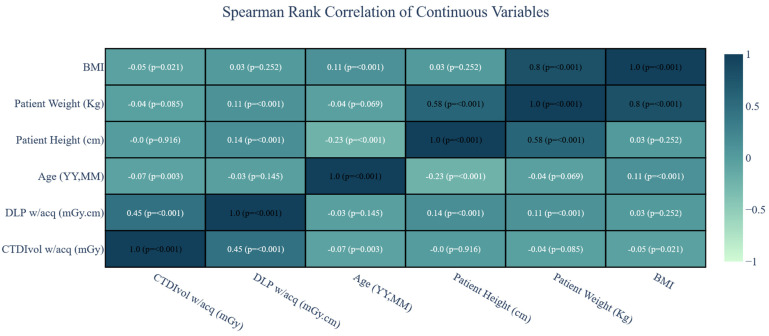
Spearman Rank Correlation of Continuous Variables.

Since the CTDIvol w/acq and DLP w/acq values were the same in both groups (as shown in Table 1 and Table 2), we included the entire group in the correlation analysis without dividing them into contrast- and non-contrast studies. Spearman’s rank correlation showed a lack of practically significant relationship between patient BMI, height, and weight, and the values of CTDIvol w/acq and DLP w/acq. This indicates that, in the case of head CT examinations, when determining local reference levels, patient physical factors can be disregarded (Figure 1).

**Figure 2:**
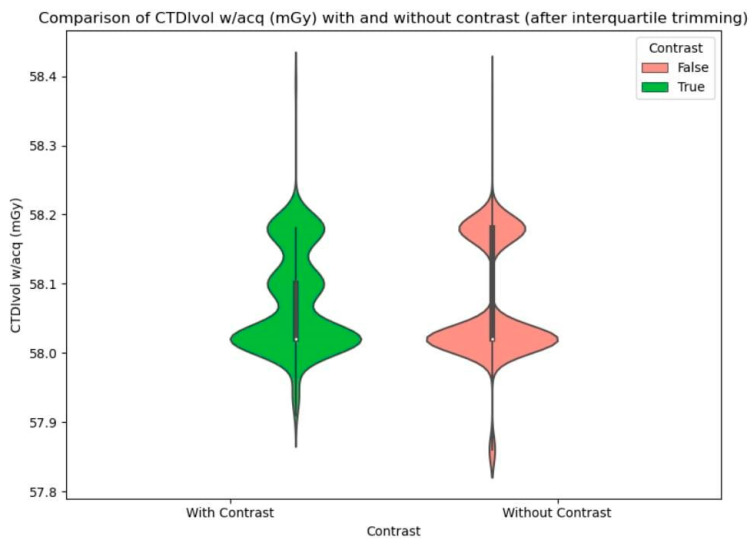
Comparison of CTDIvol w/acq (mGy) with and without contrast (after interquartile trimming). A clearly visible distribution with two subpopulations that have normal distributions.

**Figure 3:**
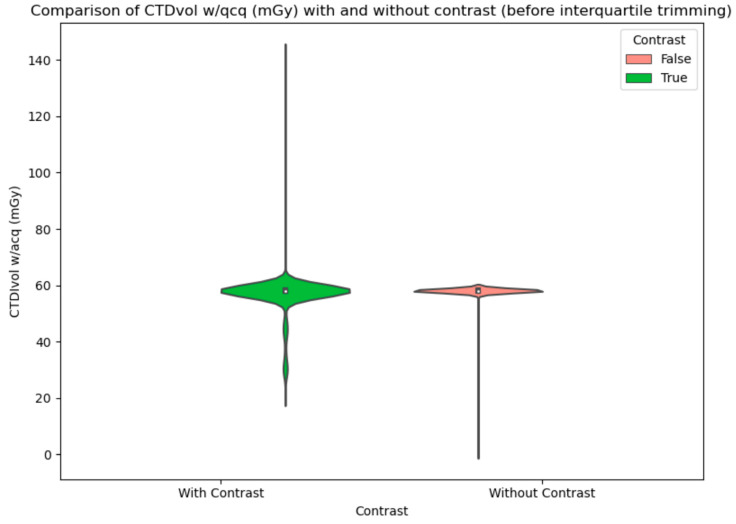
Comparison of CTDIvol w/qcq (mGy) with and without contrast (before interquartile trimming). A noticeable impact of outliers on the interpretability of the results.

**Figure 4:**
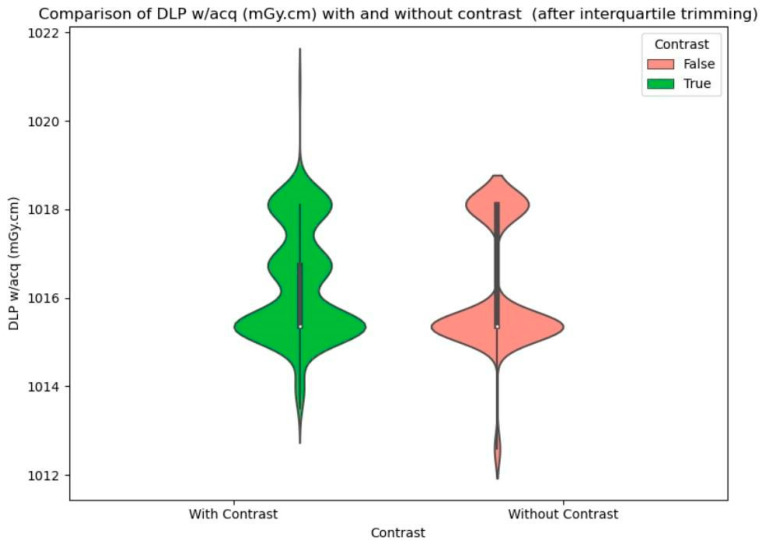
Comparison of DLP w/acq (mGy·cm) with and without contrast (after interquartile trimming).

To illustrate the difference in CTDI and DLP values for scans with and without contrast, violin plots were created before and after interquartile trimming (Figure 2, Figure 3 and Figure 4).

## 4. Discussion

The presented study aimed to establish local Diagnostic Reference Levels (DRLs) for head CT examinations, focusing on both contrast-enhanced and non-contrast scans, conducted at a university hospital in Poland. The methodology involved a retrospective analysis of a substantial number of examinations, with consideration given to the physical differences of patients.

It is worth noting that all head CT scans are performed based on a referral and under the supervision of a radiologist. This limits the patient’s exposure to an unnecessary dose of radiation, which is emphasized in the work conducted by Fatihoglu E. et al. [4].

In the Irish study conducted by W S Tan et al., dose data were collected retrospectively, and typical values for six CT head indication-based protocols were established using a sample size of 50 patients for each protocol. The typical values were set as the median of the distribution curve, and dose distributions for each protocol were compared to ascertain significant dose differences between them. The results indicated significant differences in most typical value pairings, except for certain protocols with similar scan parameters. Additionally, the study highlighted gender-based differences in dose levels, with males generally recording higher doses than females for all protocols. Comparing the findings of the Irish study with our own, some notable differences and similarities emerge. While both studies aimed to establish DRLs for head CT examinations, the methodologies differed slightly. In the Irish study, DRLs were established based on indication-based protocols, whereas our study focused on establishing DRLs based on the physical parameters of the patients. Furthermore, the findings of the Irish study revealed significant disparities in dose levels across genders for specific protocols. The statistical analysis of our study indicates that when determining local reference levels for head CT examinations, the physical factors of patients may be overlooked [8].

Another similar study, which focused on establishing DRLs of CT was carried out by Yasuhiro Fukushima et al., using the dose-length product (DLP) as the metric. Data collection involved sending datasheets to all hospitals and clinics with CT scanners in Gunma prefecture, with data obtained for patients undergoing CT during a single month (June 2010). DLP distributions were evaluated for different anatomical regions and patient age groups, with the DRL defined as the 25th and 75th percentiles of DLP. While the study highlighted variations in DLP for different anatomical regions and age groups, it particularly noted higher DLP for pediatric head CTs in Japan compared to DRLs reported from the EU or Syria. Comparing this study to our own research on establishing local DRLs for head CT examinations in Poland, it is worth noting that both studies share the overarching goal of optimizing CT parameters to minimize radiation exposure. However, the methodologies and focus areas differ significantly. In the Japanese study, DRLs were established based on DLP distributions across different anatomical regions and age groups, encompassing a broader scope of CT examinations beyond head CTs alone. This contrasts with our study, which specifically targeted head CT with a focus on physical parameters in DRL determination. One notable difference between the two studies is the approach to DRL definition. While the Japanese study defined DRLs based on the 25th and 75th percentiles of DLP distributions, our study adopted the 75th percentile of both CTDIvol and DLP values for DRL determination. This discrepancy in DRL definition methodologies may influence the comparability of results between the two studies. In terms of implications for practice, both studies underscore the importance of optimizing CT parameters to minimize radiation exposure while maintaining diagnostic quality. The Japanese study highlights the need for further investigation and optimization, particularly for pediatric head CTs, where DLP tended to be higher compared to international DRLs. Similarly, our study emphasizes the significance of establishing local DRLs tailored to specific patient populations and examination protocols to ensure optimal radiation dose management [9].

The study from Nigeria conducted by D Joseph Zira et al. focused on establishing indication-based Diagnostic Reference Levels (DRLCI) for pediatric head computed tomography (CT) examinations, a crucial endeavor given the heightened vulnerability of pediatric patients to radiation-induced cancer. A comparison between this study and our research on establishing local DRLs for adult head CT examinations in Poland reveals interesting insights into dose optimization strategies and the impact of patient demographics on DRL determination. Firstly, the Nigerian study’s findings highlight dose variations across different age groups, emphasizing the importance of tailoring radiation doses to pediatric patients’ specific age ranges. In contrast, our study primarily focused on adult head CT examinations, where age-related dose variations are less pronounced due to the relative consistency in adult anatomy and physiology. This distinction underscores the need for age-specific DRLs in pediatric imaging contexts, a consideration not directly applicable to adult imaging protocols. Regarding results comparison, both studies aimed to establish DRLs based on dose metrics such as CTDIvol and DLP. However, the specific values obtained differed significantly, reflecting variations in patient populations, imaging protocols, and institutional practices. For instance, DRLCI values for indications such as hydrocephalus and intracranial space-occupying lesions varied across different age groups in the Nigerian study. Our research focused on differences in DRLs based on the physical parameters of patients for adult head CT examinations [10].

In comparing the findings of our study to the Canadian environmental scan of Diagnostic Reference Levels (DRLs) for head CT scans, several key insights emerge. Both studies focus on establishing DRLs based on patient radiation dose metrics. In Canada, the national DRL for head CT scans is set at 1302 mGy·cm, while our study in Poland determined a local DRL of 1018 mGy·cm. The variance between these values underscores the influence of regional practices and equipment settings. Despite these differences, both studies align on the importance of setting DRLs at the 75th percentile to optimize radiation exposure while ensuring diagnostic quality [11].

Our study focused on establishing local DRLs for adult head CT examinations in one of the University Hospitals in Poland based on the physical parameters of the patients, while also considering their gender and whether the CT was conducted with contrast or not. Furthermore, comparisons with studies from Ireland, Japan, and Nigeria shed light on different methodologies and considerations for DRL determination across different populations and imaging contexts. These comparative insights underscore the importance of tailored dose optimization strategies and emphasize the need for ongoing research to ensure radiation safety and diagnostic efficacy in clinical practice.

## 5. Conclusions

Based on the conducted analysis, identical values were obtained in the reference patient groups as well as in the entire study group. Additionally, the correlation analysis did not reveal any relationship between CTDIvol w/acq and DLP w/acq with patient weight (rS = 0.04, *p* = 0.085; rS = 0.11, *p* < 0.001) and height (rS = 0.0, *p* = 0.916; rS = 0.14, *p* < 0.001). Therefore, one DRL value independent of the patient was adopted for both contrast-enhanced and non-contrast examinations, as presented in Table 3. It is noteworthy that the DRL values for the variable CTDIvol w/acq and DLP w/acq are the same, regardless of the procedure applied.

## Figures and Tables

**Table 1:** The DRL values for contrast-enhanced head CT scans in all analyzed groups—75th percentile.

Variable	Reference Group of Women (n = 187)	All Women Group (n = 248)	Reference Group of Men (n = 155)	All Men Group (n = 161)	DRL Level Regardless of Gender in the Reference Patient Group (n = 342)	DRL Level Regardless of Gender in the Entire Study Group (n = 409)
CTDIvol w/acq (mGy)	58.18	58.18	58.18	58.18	58.18	58.18
DLP w/acq (mGy·cm)	1018	1018.11	1018	1018	1018.11	1018.11
Total DLP (mGy·cm)	2046	2026.09	2047	2046.58	2046.35	2046.09

**Table 2:** The DRL values for non-contrast head CT scans in all analyzed groups—75th percentile.

Variable	Reference Group of Women (n = 187)	All Women Group (n = 855)	Reference Group of Men (n = 155)	All Men Group (n = 749)	DRL Level Regardless of Gender in the Reference Patient Group (n = 342)	DRL Level Regardless of Gender in the Entire Study Group (n = 1604)
CTDIvol w/acq (mGy)	58.18	58.18	58.18	58.18	58.18	58.18
DLP w/acq (mGy·cm)	1018	1018.11	1018	1018.11	1018.11	1018.11
Total DLP (mGy·cm)	1028	1027.98	1028	1028.47	1027.99	1027.99

**Table 3:** The DRL value for contrast-enhanced head CT scans and non-contrast head CT.

Variable	Head CT with Contrast	Head CT Without Contrast
CTDIvol w/acq (mGy)	58.18	58.18
DLP w/acq (mGy·cm)	1018.11	1018.11
Total DLP (mGy·cm)	2046.09	1027.99

## Data Availability

The original contributions presented in the study are included in the article, further inquiries can be directed to the corresponding author.
